# Socio-economic determinants of maternal health care utilization in Kailahun District, Sierra Leone, 2020

**DOI:** 10.1186/s12884-022-04597-z

**Published:** 2022-04-01

**Authors:** Desmond Maada Kangbai, Delia Akosua Bandoh, Alexander Manu, Joetrice Yewah Kangbai, Ernest Kenu, Adolphina Addo-Lartey

**Affiliations:** 1 Ghana Field Epidemiology and Laboratory Training Program, University of Ghana of School of Public Health, Legon, Accra, Ghana; 2 Department of Epidemiology and Disease Control, School of Public Health, University of Ghana, Legon, Accra, Ghana; 3 Healthcare Nursing University of Leeds, Leeds, UK

**Keywords:** Determinants, Maternal healthcare, Healthcare utilization, Skilled birth attendant, Antenatal care, Postnatal care, Kailahun, Sierra Leone

## Abstract

**Background:**

Ascertaining the key determinants of maternal healthcare service utilization and their relative importance is critical to priority setting in policy development. Sierra Leone has one of the world’s highest maternal death ratios in the context of a weak health system. The objectives of this study were to determine; the level of utilization of Antenatal Care (ANC), Skilled Delivery Attendants (SDA), Postnatal Care (PNC) services, and factors that influence the utilization of these services.

**Methods:**

We conducted a community-based cross-sectional study involving 554 women of reproductive age (15–49 years) who had at least one delivery in the last 3 years and lived in the Kailahun District, Sierra Leone from November 2019 to October 2020. Data were analysed using analysed using bivariate, multivariate and multinomial logistic regression models.

**Results:**

The median age of respondents was 25 years (Q1 = 17 years, Q3 = 30 years). Eighty-nine percent (89%) had 4 or more ANC visits. Only 35.9% of women were delivered by SDA. Women residing in urban areas had over six-fold increased odds of utilizing SDA as compared to women residing in rural areas (AOR = 6.20, 95% CI = 3.61–10.63). Women whose husbands had a primary level of education had 2.38 times increased odds of utilizing SDA than women whose husbands had no education (AOR = 2.38, 95% CI = 1.30–4.35). Women that walked longer distances (30–60 min) to seek healthcare had 2.98 times increased odds of utilizing SBA than those that walked shorter distances (< 30 min) (AOR = 2.98, 95% CI = 1.67–5.33). Women who had a secondary/vocational level of education had 2.35 times increased odds of utilizing the standard PNC category as compared to those with no education (OR = 2.35, 95% CI = 1.19–4.63).

**Conclusion:**

The majority of women had 4 or more ANC visits yet the use of skilled birth attendants was low. Urban residence and education were significantly associated with the use of the standard PNC category. To improve the utilization of maternal health care services, national healthcare policies should target the advancement of education, train skilled Maternal Healthcare (MHC) attendants, rural infrastructure, and the empowerment of women.

**Supplementary Information:**

The online version contains supplementary material available at 10.1186/s12884-022-04597-z.

## Introduction

Maternal health care (MHC) utilization is essential for women’s health, childbirth, and the well-being of the mother and child. Maternal health care includes the care a woman receives throughout her pregnancy, labor, and postnatal [1]. The World Health Organisation has set a goal in alignment with the 3rd Sustainable Development Goals (SDG) which aims to reduce the global maternal mortality ratio (MMR) to less than 70 per 100,000 live births by 2030″ [2]. To achieve this goal, ascertaining the determining factors of maternal health care utilization and how these factors affect the livelihood of mothers is critical and must be a priority in policy development [3].

Globally, 810 women die per day owing to pregnancy-related complications [4]. Maternal death remains a great concern with virtually 99% of all maternal deaths occurring in developing nations, but the prevalence is higher in sub-Saharan Africa [5]. Sierra Leone has one of the world’s highest maternal death ratios at 1360 deaths per 100,000 babies born. WHO estimated that up to 6% of women in Sierra Leone will die from maternal causes during their reproductive life” [2].

Research also suggests that this high prevalence of maternal, neonatal, and child death are linked to non- or poor availability of quality maternal healthcare services [6, 7]. Again, in sub-Saharan Africa, the factors that influence the increase in maternal mortality are also associated with prenatal care coverage, skilled attendance at delivery and postnatal care. The non-use of these maternal healthcare services is a key predictor of perinatal mortality [2].

Accessing skilled care before, throughout pregnancy, and after delivery can therefore save the lives of mothers and their newborn child infants [7, 8]. If mothers have access to healthcare facilities that provide interventions and preventive measures to treat obstetric complications, especially in an emergency, then an estimated 74% of maternal mortality could be prevented [7]. Antenatal care, skilled delivery attendants, and postnatal care attendants therefore remain key providers to improve health outcomes for mothers and their babies [9]. Studies in Ethiopia, Ghana, and Cambodia showed similar findings [6, 10, 11]. Uptake of these MCH services enables the healthcare providers to have a better chance to detect and reduce any risk factors associated with adverse pregnancy outcomes and also serves as a platform to counsel the mothers on why a skilled attendant is crucial at delivery [12, 13].

Though the use of maternal health care services offers better health outcomes for mothers and their children [14], only 45% of women using skilled delivery attendants at delivery in sub-Saharan Africa [5] and up to 13% of women in Sierra Leone still using unskilled delivery attendants [11]. There is the need to explore factors that influence the use of MCH services such as the effect of demographic factors on service utilization in Sierra Leone. Effects of these factors remains unclear, especially in districts far from the national capital such as Kailahun district, the epicenter of the 2014 Ebola outbreak. Again, the Sierra Leone Demographic and Health Survey (SLDHS) covers some components of MHC service, but details on specific factors influencing utilization are not fully addressed. Again, other previous studies conducted in Sierra Leone focused on one or two of them [15, 16].

This research sought to investigate the socio-economic factors that influence women’s use of maternal healthcare services in Eastern Sierra Leone with a focus on ANC, delivery care, and postnatal care. It will provide a further understanding of factors influencing uptake of maternal health services in Kailahun, Sierra Leone, thus helping in implementing policies that would improve maternal and child health.

## Methods

### Study design, population, and setting

We conducted a community-based cross-sectional study that involved women of reproductive age (15–49 years), who had at least one delivery in the 3 years before this study in the Kailahun District, Eastern Sierra Leone from November 2019 to January 2020. Kailahun District is located about 450 km from Freetown (the capital) in the Eastern province of Sierra Leone. Its capital and largest city is the town of Kailahun. It is divided into 15 chiefdoms. Healthcare facilities are divided into 15 community health centers (CHC), 52 community health posts (CHP), 17 maternal child health posts (MCHP), 3 government hospitals, and 2 private clinics. The total projected population for 2020 is 625,500 [17]. The total fertility rate is estimated at 6 children per woman [17]. The ANC attendance for ANC1 and ANC4+ was 72.5 and 63.9% respectively [14]. The District of Kailahun shares borders with the Republic of Liberia to the East, and the Republic of Guinea to the North.

### Sampling method

The sample size was determined using Cochran’s formula [15] with the following assumptions: the estimated proportion (*p*) of births by SBA was 0.6, the estimated proportion of births by an unskilled attendant (*q*) was 0.4, and a design effect (DEFF) of 1.5 from a previous study conducted in Sierra Leone [16]. The level of precision (*d*) was 0.05, and a 95% CI (z) was 1.96, were used to obtain a sample size of 554.

$$\mathrm{n}=\frac{z^2 pq}{d^2}\ \mathrm{X}\ \mathrm{DEFF}$$

For sampling of households, the 2015 census EAs were used as the Primary Sampling Unit (PSU) with the Urban and Rural areas of Kailahun District as the domains. The proportional allocation of household in the Kailahun District based on the domain was 69% rural and 31% urban.

A Random-walk technique was used in selecting households/respondents. At the center of the EA, the interviewer identified the third house from his right-hand side. Upon arrival in the HH only one eligible female 15–49 years had given birth in the past 3 years before this study was enrolled. Where there was more than one woman eligble, women balloted by picking yes or no on small sheets of paper. The interviewer moved in the eastward direction into every third house. If no one is eligible for a particular HH, we continued with the pattern of selecting the HH.

### Data collection technique and tools

The primary data was collected using a structured and pre-tested questionnaire. Antenatal cards and hospital record books of mothers were inspected to confirm the information provided by women during the interview. The questionnaire was written in English and was adapted from the Standard Sierra Leone DHS Questionnaires [16].

### Data quality management

To ensure quality data, interviewers were trained, a pre-test was performed before the actual data collection. A supervisor was assigned to interviewers throughout the data collection period to ensure data was collected appproriately. Daily spots checks of filled questionnnaires was done and issues itendified resolved before the day’s work began. Data validation checks were done to ensure accuracy during data entry.

### Operational definitions

- Skilled delivery attendant: professionals with midwifery skills including Doctors, midwives, community health officers (CHO).
- Antenatal care: Care provided by skilled healthcare professionals to pregnant women and adolescent girls in other to ensure the best health conditions for both mother and baby during pregnancy.
- Postnatal care: services provided to women within 42 days after delivery by health professionals.

### Outcome variables

A set of questions on maternal healthcare, covering place of delivery, delivery attendant, place of PNC, PNC attendant, the timing of the first postnatal visit, follow-up after discharge, and place of follow-up after discharge were asked. For the study, the outcome variable which was the utilization of maternal health care services was assessed by two services:

- Skilled Delivery Attendant use (yes/no)
- Postnatal care service (Standard, Average and Undesirable):

1. Standard category: PNC from a skilled provider in a health facility within 1 h after delivery; had at least one follow-up after discharge.
2. Average category: PNC from a non-skilled provider in a health facility within 1 h after delivery and had at least one follow-up after discharge or PNC from a skilled provider but did not receive any of the other components- health facility delivery; no follow-up after discharge.
3. Undesirable category: Postnatal care from a non-skilled provider in a non-health facility, no follow-up after discharge.

### Independent variables

Andersen’s behavioral model was adopted to determine the influence or effect of the independent variables (predisposing and enabling factors) on the utilization of maternal health services [18, 19]. The predisposing factors were age, women’s education, parity, ethnicity, religion, marital status, occupation, number of births in the last 5 years, number of children alive, and the enabling factors were husbands education, residence, distance to the health facility, type of floor and toilet.

### Data analysis

The data were entered into MS Excel 2018 and cleaned. The analysis was done using Stata IC 15.0, College Station, TX: StataCorp LLC. Frequencies and proportion were generated to describe the Predisposing and Enabling characteristics, the components/patterns of antenatal care, perinatal care/skilled delivery attendant, and postnatal care. Frequencies were presented as tables. Bivariate analysis was done to determine the level of utilization of Maternal Healthcare services (Antenatal care, Skilled delivery attendant, and Postnatal care services).

In the case of a skilled delivery attendant, a dichotomous dependent variable was constructed to indicate whether or not the woman used services from a skilled provider. Bivariate and multivariate analysis techniques were used to estimate the nature of association and magnitude between dependent variables and independent variables (Individual and Enabling factors).

Initially, a simple binary logistic model was run for each independent variable against the outcome variable. All independent variables that showed a significant association at *p* < 0.05 were included in the multivariate binary regression model. The *P*-value, crude odds ratios (COR), adjusted odds ratios (AOR), and 95% confidence interval were estimated.

### Ethical clearance

Ethical approval was obtained from the Sierra Leone Ethics and Scientific Review Committee (October 2019). Permission was also sought from the Kailahun Regional Health Directorate. Informed consent was obtained from the respondents. For respondents below 18 years, consent was granted by the parent or guardian, and assent was sought from respondents. They were assured of confidentiality and informed of the purpose of the study. The information was stored without the names of the respondents in a folder that is only accessible to the data team. For participants who could not read, the consent was read and explained to them in the presence of an impartial witness or stakeholder in the community who also appended his signature on the consent form in accordance with the local IRB regulation. All study methods were carried out following relevant guidelines and regulations.

## Results

### Background characteristics of respondents

Five hundred and fifty-four (554) women aged between 15 and 49 years, who had at least a delivery/birth 3 years before this study commenced were interviewed. The median age of respondents was 25 years with minimum and maximum ages of 15 and 49 years respectively (Table 1). Taking into consideration parity, the median parity was 3 with a minimum and maximum parity of 1 and 9 respectively. Forty-six percent (46%) of respondents in this survey were between the ages of 25–35 years and 37.9% had no educational level. The majority (89.5%) of respondents were either married or living together with a partner, 6.7% were single and 3.8% were divorced/Separated/widowed. Most of the respondents (68.8%) were rural residents. The majority (60.8%) of respondents were Muslims. The Mende ethnic group was predominant, (72%).

**Table 1:** Enabling and predisposing characteristics of respondents who had at least one delivery in the 3 years before the study, Kailahun District, Sierra Leone, 2020

Predisposing/Enabling Characteristics	Number (***n*** = 554)	Percent(%)
Age		
10–19	76	13.7
20–24	172	31.0
25–35	254	45.8
> 35	52	9.4
Schooling		
No	210	37.9
Yes	344	62.1
Education level attainment		
None	210	37.9
Primary	142	25.6
Junior Secondary	142	25.6
Senior Secondary	56	10.1
Vocational/Higher	4	0.7
Religion		
Christian	217	39.2
Islam	337	60.8
Ethnicity		
Mende	399	72.0
Kissi	124	22.4
Others	31	5.6
Number of Children Alive		
0–2	275	49.6
3–5	231	41.7
6+	48	8.7
Parity		
1	132	23.8
2–4	292	52.7
5+	130	23.5
Number of Births in the past 5 years		
1	265	47.8
2+	289	52.2
Marital Status		
Single	37	6.7
Married/Living Together	496	89.5
Divorced/separated/Widowed	21	3.8
Occupation		
Employed	433	78.2
Unemployed	121	21.8
Distance to Health Facility (Minutes)		
< 30	196	35.4
30–60	132	23.8
> 60	226	40.8
Husband’s Occupation		
Unemployed	78	14.1
Employed	476	85.9
Husband’s Education level attainment		
None	238	43.0
Primary	72	13.0
Junior Secondary	109	19.7
Senior Secondary	82	14.8
Vocational/Higher	53	9.6
Residence		
Rural	381	68.8
Urban	173	31.2
Type of Floor		
Natural	394	71.1
Modern	160	28.9
Type of Toilet		
Flush	14	2.5
Pit/Others	540	97.5

### The components of ANC, skilled delivery, and PNC utilization

The majority of women (77.6%; 430/554) have had ANC from a skilled provider. Most of the women (57.9%; 321/554) received ANC from a State Enrolled Community Health Nurse (SECHN). Regarding the timing of the first ANC visit, (54.5%; 302/554) of respondents attended their first ANC visit in the first trimester. The majority of respondents (88.6%; 491/554) had 4 or more ANC visits in their last pregnancy before this study (Table 2).

**Table 2:** Characteristics of MHC service utilization by respondents who had at least one delivery in the 3 years before the study, Kailahun District, 2020

Variable	Number (***n*** = 554)	Percent(%)
At least one ANC from Skilled Provider		
No	124	22.4
Yes	430	77.6
ANC Provider		
Traditional Birth Attendant	26	4.7
MCHA	98	17.7
Midwife	100	18.1
Doctor	2	0.4
Nurse	321	57.9
CHO	7	1.3
Timing of First ANC		
Don’t Know	15	2.7
< 4 months	302	54.5
4–6 months	234	42.2
7–9 months	3	0.5
Number of ANC visit		
Do not know	5	0.9
Once	4	0.7
2 times	11	2.0
3 times	43	7.8
4 or more	491	88.6
Skilled Delivery Attendant		
No	355	64.1
Yes	199	35.9
Delivery Attendant		
TBA (health facility/ community)	23	4.2
Nurse	13	2.3
MCHA	319	57.6
Midwife	177	31.9
Doctor	22	4.0
Place of Delivery		
MCHP	58	10.5
CHP	199	35.9
CHC	198	35.7
Hospital	75	13.5
Private Facility	20	3.6
Home	3	0.5
Ambulance/Transit	1	0.2
Postnatal Care		
Yes	540	97.5
No	14	2.5

### Socio-demographic characteristics and the use of MHC

The level of utilization of skilled ANC attendants was more common among respondents aged > 35 years compared to the other age groups. In general, the use of skilled ANC attendants was 75.0% or more among all background characteristics assessed. With regards to parity, as the parity increased the use of skilled ANC attendants decreased, more women with no education attainment received at least one ANC visit (78.6%) compared to educated women (77.5%).

The use of SDA was found to be higher (48.7%) amongst single compared with married women 34.7% (62/172).

Similar to ANC use, the use of skilled birth attendants decreased as the parity increased, 43.2% (57/132) of women who had 1 parity used skilled birth attendants compared with 37.3% (226/292) of those who had 2–4 parities and 25.4% of those who had 5 or more births.

More than 90% of all women interviewed received PNC services. All women whose husbands were unemployed received PNC services. In contrast to ANC and skilled birth attendant use, PNC service use was slightly higher (97.9%; 372/381) among women in rural areas than women in urban areas (96.5%; 167/173) (Table 3).

**Table 3:** Socio-demographic characteristics and the use of MHC Services among women in Kailahun District, Sierra Leone, 2020

Background Characteristics	Number (***n*** = 554)	Percentage who received at least one ANC	Percentage who received SDA	Percentage who received PNC services
Respondents	***554***	***77.6***	***35.9***	***97.5***
Age				
10–19	76	76.3	42.1	97.4
20–24	172	77.9	36.1	98.3
25–35	254	76.4	35.4	97.2
> 35	52	84.6	28.9	96.2
Residence				
Urban	173	81.5	57.8	96.5
Rural	381	75.9	26	97.9
Marital Status				
Single	37	78.4	48.7	100
Married/Living Together	496	77.2	34.7	97.2
Divorced/Separated/Widowed	21	85.7	42.9	100
Religion				
Christian	217	79.7	37.3	98.6
Islam	337	76.3	35	96.7
Ethnicity				
Mende	399	76.2	35.3	97.5
Kissi	124	80.7	37.9	98.4
Others	31	83.9	35.5	93.5
Parity				
1	132	79.6	43.2	97.7
2–4	292	77.4	37.3	97.9
5+	130	76.2	25.4	96.2
Occupation				
Employed	433	81.8	48.8	99.2
Unemployed	121	76.4	32.3	97
Education level attainment				
None	210	78.6	30.9	96.7
Primary	142	77.5	38	98.6
Junior Secondary	142	71.8	33.8	96.5
Senior Secondary/Vocational	60	88.3	53.3	100
Number of Births in the past 5 years				
1	265	80.4	39.6	97.7
2+	289	75.1	32.5	97.2
Husband’s Occupation				
Unemployed	78	79.5	44.9	100
Employed	476	77.3	34.5	97.1
Husband’s Education level attainment				
None	238	75.2	30.3	97.9
Primary	72	79.2	44.4	97.2
Junior Secondary	109	80.7	37.6	98.2
Senior Secondary	82	78.1	30.5	97.6
Vocational/Higher	53	79.3	54.7	94.3
Distance to Health Facility (Minutes)				
< 30	196	77	35.7	96.4
30–60	132	75	47	98.5
> 60	226	79.7	29.7	97.8
Type of Floor				
Natural	394	77.4	34	97.2
Modern	160	78.1	40.6	98.1
Type of Toilet				
Flush	14	85.7	35.7	100
Pit and Others	540	77.4	35.9	97.4

### Background characteristics influencing the use of a skilled ANC attendant

Maternal age was not significantly linked with the use of skilled ANC providers. Women aged > 35 years were more likely to use a skilled ANC attendant than women aged 10–19 years which was found not to be significant (COR 1.71, 95% CI 0.68–4.28). There was no significant association between marital status and the use of a skilled ANC provider. The odds of a skilled ANC attendant use were 1.66 times higher in divorced/separated/widowed compared to single women which were not statistically significant (COR 1.71, 95% CI 0.68–4.28). There was no significant association between religion, ethnicity, occupation, education, and the use of skilled ANC providers (Table 4).

**Table 4:** Factors associated with the use of skilled ANC attendant, Kailahun District, Sierra Leone, 2020

Variable	ANC		Crude OR (95% CI)
	Yes *n* = 430(%)	No *n* = 124(%)	
Age			
10–19	58(13.5)	18(14.5)	1.00
20–24	134(31.2)	38(30.7)	1.09(0 .58 2.08)
25–35	194(45.1)	60(48.4)	1.00(0.55 1.83)
> 35	44(10.2)	8(6.5)	1.71(0.68 4.28)
Residence			
Rural	289(67.2)	92(74.2)	1.00
Urban	141(32.8)	32(25.8)	1.40(0 .89 2.20)
Religion			
Christain	173(40.2)	44(35.5)	1.00
Islam	257(59.8)	80(64.5)	0.82(0.54 1.24)
Ethnicity			
Others	26(6.05)	5(4.0)	1.00
Mende	304(70.7)	95(76.6)	0.62(0.23 1.65)
Kissi	124(22.4)	24(19.4)	0.80(0.28 2.30)
Parity			
1	105(24.4)	27(21.8)	1.00
2–4	226(52.6)	66(53.2)	0.82(0.53 1.46)
5+	99(23.0)	31(25.0)	0.88(0 .46 1.47)
Education level attainment			
None	165(38.4)	45(36.3)	1.00
Primary	110(25.60	32(25.8)	0.94(0.56 1.57)
Junior Secondary	102(23.70	40(32.3)	0.70(0.43 1.14)
Senior Secondary/Vocational	53(12.3)	7(5.7)	2.06(0.43 1.14)
Number of Births in past 5 years			
1	213(49.5)	52(41.9)	1.00
2+	217(50.5)	72(58.1)	0.74(0.49 1.10)
Husband’s Education level attainment			
None	179(41.6)	59(47.6)	1.00
Primary	57(13.3)	15(12.1)	1.25(0.66 2.38)
Junior Secondary	88(20.5)	21(16.9)	1.38(0.79 2.42)
Senior Secondary	64(14.9)	18(14.5)	1.17(0.64 2.14)
Vocational/Higher	42(9.8)	11(8.9)	1.25(0.61 2.60)
Distrance to Health Facility (Minutes)			
< 30	151(35.1)	45(36.3)	1.00
30–60	99(23.2)	33(26.6)	0.89(0.53 1.50)
> 60	180(41.9)	46(37.1)	1.17(0.73 1.86)
Type of Floor			
Natural	305(70.9)	89(71.8)	1.00
Modern	125(29.1)	35(28.3)	1.04(0.67 1.62)
Type of Toilet			
Pit and Others	418(97.2)	122(98.4)	1.00
Flush	12(2.8)	2(1.6)	1.75(0.39 7.93)

### Background characteristics influencing the use of a skilled delivery attendant

Place of residence, parity, occupation, educational attainment, husband educational attainment, distance to health facility were significantly associated to skilled delivery attendant (Table 5). Women residing in urban areas had over six-fold increased odds of being delivered by a skilled attendant as compared to women residing in rural areas. This association remained statistically significant even after controlling for the effect of the other variables (AOR = 6.20, 95% CI = 3.61–10.63). Women who had a senior secondary or vocational level of education had 1.22 times increased odds of being delivered by a skilled attendant as compared to those with no educational level which was statistically not significant after controlling for the effect of other variables (AOR = 1.22, 95% CI = 0.57–2.59).

**Table 5:** Factors associated with the use of skilled delivery attendant, Kailahun District, Sierra Leone, 2020

Variable	SDA		COR (95% CI)	AOR (95% CI)
	No ***n*** = 355(%)	Yes ***n*** = 199(%)		
Age				
10–19	44(12.4)	32(16.1)	1.00	
20–24	110(31.0)	62(31.2)	0.78(0.45 1.35)	.
25–35	164(46.2)	90(45.2)	0.75(0.45 1.27)	.
> 35	37(10.4)	15(7.5)	0.56(0.26 1.18)	.
Residence**				
Rural	282(79.4)	99(49.8)	1.00	1.00
Urban	73(20.6)	100(50.3)	3.90(2.67 5.70)*	6.20(3.61 10.63)*
Parity**				
1	75(21.1)	57(28.6)	1.00	1.00
2–4	183(51.6)	109(54.8)	0.78(0.52 1.19)	0.90(0.56 1.47)
5+	97(27.3)	33(16.6)	0.45(0.27 0.76)*	0.58(0.31 1.10)
Religion				
Christian	136(38.3)	81(40.7)	1.00	.
Islam	219(61.7)	118(59.3)	0.90(0.63 1.29)	.
Marital Status				
Single	19(5.4)	18(9.1)	1.00	.
Married/Living Together	324(91.3)	172(86.4)	0.56(0.29 1.10)	.
Divorced/Separated/Widowed	12(3.4)	9(4.5)	0.79(0.27 2.33)	.
Ethnicity				
Others	20(5.6)	11(5.5)	1.00	.
Mende	258(72.7)	141(70.9)	0.99(0.46 2.13)	.
Kissi	77(21.7)	47(23.6)	1.11(0.49 2.52)	.
Occupation**				
Unemployed	62(17.5)	59(29.6)	1.00	1.00
Employed	293(82.5)	140(70.4)	0.50(0.33 0.76)*	0.67(0.40 1.13)
Education level attainment**				
None	145(40.9)	65(32.7)	1.00	1.00
Primary	88(24.8)	54(27.1)	1.37(0.87 2.14)	0.97(0.58 1.62)
Junior Secondary	94(26.5)	48(24.1)	1.14(0.72 1.79)	0.91(0.53 1.58)
Senior Secondary/Vocational	28(7.9)	32(16.1)	2.25(1.42 4.58)*	1.22(0.57 2.59)
Number of Births in past 5 years				
1	160(45.1)	105(52.8)	1.00	.
2+	195(54.9)	94(47.2)	0.73(0.52 1.04)	.
Husbands Occupation				
Unemployed	43(12.1)	35(17.6)	1.00	.
Employed	312(87.9)	164(82.4)	0.65(0.40 1.05)	.
Husband Education level attainment**				
None	166(46.8)	72(36.2)	1.00	1.00
Primary	40(11.3)	32(16.1)	1.84(1.07 3.17)*	2.38(1.30 4.35)*
Junior Secondary	68(19.2)	41(20.6)	1.39(0.86 2.24)	1.34(0.79 2.29)
Senior Secondary	57(16.1)	55(12.6)	1.01(0.59 1.74)	0.67(0.35 1.25)
Vocational/Higher	24(6.8)	29(14.6)	2.79(1.52 5.11)*	1.85(0.93 3.72)
Distance to Health Facility** (Minutes)				
< 30	126(35.5)	70(35.2)	1.00	1.00
30–60	70(19.7)	62(31.2)	1.59(1.02 2.50)*	2.98(1.67 5.33)*
> 60	159(44.8)	67(33.7)	0.76(0.50 1.14)	2.37(1.33 4.24)*
Type of Floor				
Natural	260(73.2)	134(67.3)	1.00	.
Modern	95(26.8)	65(32.7)	1.33(0.91 1.94)	.
Type of Toilet				
Pit and Others	346(97.5)	194(97.5)	1.00	.
Flush	9(2.5)	5(2.5)	0.99(0.33 3.00)	.

*Significant association at *P* < 0.05 ** Variables adjusted for the effect of the other variables

### Background characteristics influencing the use of postnatal care services

The level of education of a woman, residence, and the husband’s education level, were significantly associated with the use of the standard PNC category relative to the average category after controlling for the effect of the other variables in the model (Table 6). Women who had a secondary/vocational level of education had 2.35 times increased odds of utilizing the standard PNC category as compared to those with no education relative to the average PNC category (COR = 2.35, 95% CI = 1.19–4.63). Women’s residence was significantly associated with the use of the standard PNC category relative to the average category. Women residing in urban areas had 2.29 times increased odds of utilizing the standard PNC category as compared to those residing in rural areas (COR = 2.29, 95% CI = 1.21–4.32). Husband’s education was significantly associated with the use of the standard PNC category relative to the average category. Women whose husbands had a primary level of education had 2.36 times increased odds of utilizing the standard PNC category as compared to those whose husbands had no education relative to the average PNC category (COR = 2.36, 95% CI = 1.10–5.05).

**Table 6:** Multinomial logistic regression results on the determinants of PNC services, Kailahun District, Sierra Leone, 2020

Variables	Number (***n*** = 554)	Standard PNC		Undesirable PNC	
		COR(95% CI)	***P***-Value	COR(95% CI)	***P***-Value
Age (Continuous)	554	1.04(0.98 1.10)	0.199	0.99(0.88 1.12)	0.923
Parity	554	0.69(0.38 1.24)	0.215	1.15(0.34 3.90)	0.818
Residence					
Rural	381	1.00		1.00	
Urban	173	2.29(1.21 4.32)	0.011*	1.59(0.37 6.79)	0.535
Marital Status	554	0.82(0.38 0.76)	0.617	1.09(0.15 7.80)	0.933
Religion					
Christian	217	1.00		1.00	
Islam	337	0.71(0.41 1.23)	0.220	1.99(0.43 9.14)	0.375
Ethnicity					
Others	399	1.00		1.00	
Mende	124	1.23(1.23 0.67)	0.720	0.31(0.05 1.91)	0.207
Kissi	31	0.62(0.18 2.13)	0.453	0.26(0.02 2.76)	0.263
Occupation					
Employed	433	1.00		1.00	
Unemployed	121	1.13(0.61 2.11)	0.689	4.04(0.46 35.88)	0.210
Education level attainment					
None	210	1.00		1.00	
Primary	142	1.10(0.55 2.22)	0.780	0.35(0.06 1.90)	0.224
Secondary/Vocational	202	2.35(1.19 4.63)	0.013*	0.89(0.21 0.69)	0.872
Number of Births in past 5 years					
1	265	1.00		1.00	
2+	289	1.02(0.56 1.84)	0.958	0.90(0.26 3.10)	0.867
Husband’s Education level attainment					
None	238	1.00		1.00	
Primary	72	2.36(1.10 5.05)	0.027*	1.73(0.30 9.97)	0.537
Junior Secondary	109	1.88(0.96 3.69)	0.064	1.09(0.20 6.06)	0.923
Senior Secondary	82	0.81(0.35 1.85)	0.610	1.27(0.20 8.06)	0.802
Vocational/Higher	53	2.29(1.03 5.12)	0.042*	4.07(0.77 21.51)	0.098
Distance to Health Facility (Minutes)					
< 30	196	1.00		1.00	
30–60	132	1.36(0.71 2.62)	0.357	0.61(0.10 3.55)	0.580
> 60	226	1.42(0.72 2.81)	0.312	0.82(0.19 3.66)	0.802
Type of Floor					
Natural	394	1.00		1.00	
Modern	160	1.35(0.80 2.27)	0.266	0.49(0.12 1.99)	0.318

*Significant association at *P* < 0.05, *n* = 554, pseudo *R*^2^ = 8.81%, LR Chi2 = 54.37

Prob. Chi2 = 0.025, Base model = Average category

## Discussion

This study assessed the level of utilization and determinants of ANC, skilled birth attendant, and uptake of the different packages of PNC services in Kailahun District Sierra Leone. This study found that 100.0% of women received ANC services, of which 77.6% of them sought at least one ANC visit from a skilled ANC provider and 88.6% made 4 or more ANC visits as recommended by the World Health Organization. These findings are similar to reports in the Sierra Leone Demographic and Health Survey which showed that 98.0% of women received ANC from a skilled provider and 79.0% made 4 or more ANC visits [11]. However, our results are also inconsistent with the Uganda Demographic and Health Survey UDHS 2016, which showed that 97.0% of women received ANC from a skilled provider but only 60.0% of the women made 4 or more ANC visits [20]. The disparity between the present study and the UDHS may be related to the maternal demographic characteristics in both countries and the fact that the sample size in the UDHS was far larger, thus influencing the precision of the findings.

Regarding the timing of the first ANC, 54.5% of women sought their first ANC in the first trimester of pregnancy and this prevalence was higher than that obtained in the Sierra Leone Demographic and Health Survey 2019, where 44.0% made their first visit in the first trimester [11]. This discrepancy could be attributed to the demographic survey having had a larger coverage area as compared to the present study. It has been recommended that all pregnant women should start their ANC in the first trimester [21]. The findings of this research suggest that the use of ANC services was higher among urban residents (81.5%), which corroborates a study conducted in Holeta Town, Ethiopia where 86.7% of urban women used ANC services [22]. According to the Sierra Leone Demographic Survey (2019), 73.0% of women in urban areas made 4 or more ANC visits which is slightly lower patronage as compared to our population. The high use of ANC services in urban areas may be because, Kailahun District has 87 peripheral health units and 3 hospitals, of which all the hospitals and most of the PHUs are in urban areas thus increasing access. Increased awareness and information sharing might also be related to the economic status of urban residents since women in urban areas have more physical and economic access to health facilities.

The findings of this study showed that the enabling (maternal age, marital status, ethnicity, parity, respondent education, and religion) and predisposing factors (residence, distance to the health facility, and husband’s education) were non-predictor of ANC service utilization. A study conducted in Holeta Town, Central Ethiopia found maternal age and education as predictors of ANC service utilization [22]. In another study in Ghana, residence and education were major predictors of ANC services [1, 23, 24].

This research further indicated that the use of SDA was generally low. Only 35.9% of women had at least one delivery in 3 years before this study used SDA compared to 11.7% in a study in Ethiopia [5]. The majority, 57.6, and 4.2% were delivered by Maternal and Child Health Aide (MCHA) and Traditional Birth Attendant (TBA) respectively, who are considered non-skilled. According to the Demographic and Health Survey (2019), 87% of deliveries were assisted by a skilled provider. The disparity between the DHS and this study may be related to the fact that our study did not consider MCHA and State Enrolled Community Health Nurses (SECHN) as skilled attendants. Kailahun District is one of the most remote areas in Sierra Leone, physical accessibility is a major challenge due to the bad road network, thus most healthcare workers find it difficult to travel, work and stay in the district which creates opportunities for non-skilled workers. The WHO has recommended that there should be a critical threshold of 23 skilled healthcare workers (doctors, nurses, and midwives) per 10,000 population [25]. Nevertheless, it has been very difficult for Sierra Leone to cope with such recommendations due to the severe scarcity of qualified healthcare workers, thereby providing merely 2 skilled workers per 10,000 populations [14]. The 10 years of civil war which ended in 2002 and the 2014 Ebola epidemic, all started in Kailahun District and left a huge impact on health service delivery in the district. Sierra Leone is among the world’s highest maternal death ratios at 1360 mortality per 100,000 births [2] because most women are not delivered by a SDA, and most ANC services are provided by non-skilled providers. It further found that 10.5 and 35.9% of deliveries took place at Maternal and Child Health Post (MCHP) and Community Health Post (CHP) respectively, which are facilities manned by non-skilled attendants.

This study found that the area of residence is a major determinant of SDA utilization. The use of skilled birth attendants was higher among urban residents than rural. These findings are consistent with other studies [5, 7]. The disparity in the utilization of MHC services may be due to the concentration of health facilities in urban areas combined with the high number of qualified birth attendants in urban areas and also the economic status of the urban residents. In the Kailahun district, there is an uneven distribution of health workers, most are found in urban areas. Distance to the health facility was considerably connected with the use of SDA. Women that walked 30–60 min or more than 60 min to access health care services were more likely to use SDA than those that walked less than 30 min. In another study conducted in Kenya, although the distance was cited as a barrier to MHC service utilization, 18% of women did not visit the nearest facility [26].

In the present study, the husband’s education was significantly associated with the use of a skilled delivery attendant. Women whose husbands had at least primary education were more inclined to use SDA than those whose husbands had no education which is consistent with other studies [5]. Similarly, in another study conducted in Nigeria, husbands’ education played a key role in the utilization of SDA [1], research has shown that education increases health awareness and knowledge on the significance of MHC services and improves other forms of learning [5]. This could be through; radio, the internet, written information, and a better cultural understanding. Educated husbands may provide more autonomy to their wives [5].

Our study found that the majority 97.5% of women received PNC services and 58.1% of them received it from MCHA. The 2013 Sierra Leone Demographic and Health Survey reported that 7.8% of PNC services were delivered by MCHA [11]. The disparity between the national and district figures may be related to the fact that the Demographic Survey was done in the entire 16-district taking into consideration the major urban areas where good healthcare services are concentrated compared with Kailahun being one of the most remote districts. Also, due to the limited number of skilled providers in the district, most PNC services are provided by non-skilled providers.

In the present study, women’s residence, education of women, and husband’s education are significant predictors of the utilization of the standard PNC package. These observations are consistent with a study conducted in Ethiopia among women of reproductive age which reported a significant influence of respondent education and urban residence on the utilization of MHC services [5].

Some of the results of this study are similar to others reported, which have highlighted the importance of effective, efficient, and accessible Maternal healthcare services. Several issues specific to Sierra Leone were identified that suggest the influence of these socioeconomic factors on the utilization of MHC services and if these are not addressed in addition to the inadequate number of skilled attendants, Sierra Leone would continue to have high maternal and infant mortality ratios. Finally, our results show that investment in the training of skilled attendants, education, and rural healthcare would significantly improve the utilization of MHC service thus reducing maternal and infant mortality.

### Limitation of study

Some of the respondents seemed to have difficulty in the recollection of events that had happened during the last 3 years before the study. Such women had difficulties in recalling or identifying the nature of healthcare services they received or the trained healthcare worker that provided the service. As a way of minimizing this challenge, the interviewers requested for participants’ ANC cards to fact-check their responses.

This study is externally valid and generalizable as the sample accurately represents the population and the characteristic of Kailahun District are similar to others in Sierra Leone. The households were proportionately distributed to urban and rural areas. We did not assess the healthcare facility and the health workers’ related factors that affect antenatal, postnatal, and skilled delivery utilization, all of which are known to be key determinants of maternal health care utilization. These are areas for further study.

## Conclusion

Our findings indicate that PNC service utilization in the Kailahun district is much higher compared to skilled ANC and birth attendant utilization, even though the uptake of the standard PNC package was low. The utilization of SDA was low and urban residents seemed to be using more of the skilled birth attendant services. As recommended by WHO, the majority of women had 4 or more ANC visits, and most made their first ANC visit in the first trimester. Most of the deliveries were done by unskilled personnel. Education of women, residence, parity, occupation, husband’s education, and distance to health facility had a significant association with the use of skilled birth attendants. Finally, the findings show that urban residence and higher/vocational education are significantly associated with the uptake of the standard PNC package.

To improve the utilization of maternal health care services, the Ministry of Health and Sanitation/Central Government should work with relevant stakeholders to formulate policies and design programs that target the advancement of education, rural infrastructure, and the empowerment of women. The ministry of health should Engage the research and scientific committee to undertake or facilitate further research programs to determine the healthcare factors that influence the uptake of MHC services.

## Supplementary Information

**Additional file 1.**

## Data Availability

All data generated or analyzed during this study are included in this published article [and its supplementary information files].

## Abbreviations

ANC: Antenatal care
AOR: Adjusted odds ratio
CHC: Community health center
CHO: Community health officer
CHP: Community health post
CI: Confidence interval
COR: Crude odds ratio
DHS: Demographic and health survey
EAs: Enumeration areas
MCHA: Maternal and child health aide
MCHP: Maternal and child health post
MHCS: Maternal health care service
PNC: Postnatal care
PSU: Primary sampling unit
SDA: Skilled Delivery Attendant
